# The clinical profile of Kawasaki disease of children from three Polish centers: a retrospective study

**DOI:** 10.1007/s00296-013-2836-7

**Published:** 2013-07-28

**Authors:** Daiva Gorczyca, Jacek Postępski, Edyta Olesińska, Małgorzata Lubieniecka, Iwona Lachór-Motyka, Violetta Opoka-Winiarska, Anna Gruenpeter

**Affiliations:** 1 3rd Department and Clinic of Paediatrics, Immunology and Rheumatology of Developmental Age, Wroclaw Medical University, ul. Koszarowa 5, 51-149 Wrocław, Poland; 2 1st Department of Paediatrics Pulmonology and Rheumatology, University of Medicine in Lublin, ul. Chodźki 2, 20-093 Lublin, Poland; 3 Department of Paediatric Rheumatology, John Paul II Paediatric Centre, ul. Gabrieli Zapolskiej 3, 41-218 Sosnowiec, Poland

**Keywords:** Kawasaki disease, Children, Incomplete Kawasaki, Coronary artery aneurysms

## Abstract

Kawasaki disease (KD) is one of the most common vasculitides of childhood. The aim of this retrospective study is to determine the incidence of KD and to evaluate its presenting symptoms, clinical course, laboratory tests, and treatment in patients with complete KD and incomplete KD at three pediatric rheumatology centers in Poland from January 2011 to December 2012. A total of 27 Caucasian children (12 boys and 15 girls) with median age of 3 years (range 4 months–12 years) were included in this study. The incidence of complete versus incomplete KD was 17 (63 %) versus 10 (37 %) children, respectively. Patients with incomplete KD significantly less presented cervical lymphadenopathy (20 vs. 88.2 %; *p* = 0.00075), changes in extremities (30 vs. 76.5 %; *p* = 0.04), and bilateral nonpurulent conjunctivitis (60 vs. 100 %; *p* = 0.01). Cardiac assessments show that the majority of patients with KD have not got coronary artery aneurysms (CAA). The median time from the onset of symptoms to intravenous immunoglobulin (IVIG) infusion was 7 days for complete KD and 11 days for incomplete KD. IVIG delay in the incomplete KD had no effect on the incidence of CAA. In conclusion, there were no differences in demographic features, age of onset, and laboratory tests of patients with complete and incomplete KD. Patients with incomplete KD significantly rarely presented cervical lymphadenopathy, changes in extremities, and conjunctival injection. Electrocardiography is a sensitive test to recognize cardiac involvement in the acute phase of KD. Despite the fact that incomplete forms of presentation often delay diagnosis, in most patients treatment with IVIG can avoid complication of CAA.

## Introduction

Kawasaki disease (KD) is an acute vasculitis of the medium- and small-sized arteries of unknown etiology. It occurs worldwide and predominantly affects infants and preschool children.

In the last decades, an increase in the prevalence of KD in Japan [1], the United States [2], and Europe has been observed [3, 4]. The range of incidence of KD in European countries is 5–8/100,000 children under the age of 5 years [5, 6].

There is still no definitive diagnostic test to confirm KD. American Heart Association (AHA) in 2004 established diagnostic guidelines for the initial estimate, treatment in the acute phase, and long-term management of patients with KD [7]. Complete KD is defined by fever ≥5 days and more than or equal to four of the five principal clinical features (bilateral conjunctival injection, cervical lymphadenopathy, polymorphous skin rash, changes in the lips or oral mucosa, and changes in the distal extremities) [7]. In a case of a child with fever of unexplained origin, who does not fulfill the classic criteria, and other diseases with similar clinical features are excluded, incomplete KD is diagnosed. This diagnosis is often based on echocardiographic findings of coronary artery abnormalities [7]. Laboratory parameters in KD patients include leukocytosis with thrombocytosis, elevated erythrocyte sedimentation rate (ESR), C-reactive protein (CRP) level, transaminase level, and hypoalbuminemia [7, 8].

The current treatment strategy is based on intravenous immunoglobulin (IVIG) infusion and leads to reduction in coronary artery abnormalities [7]. Response to initial therapy, regression and/or evolution of coronary lesions, varies among individuals [9].

The purpose of the study is to determine the incidence of KD and to evaluate its presenting symptoms, clinical course, particularly cardiovascular involvement, laboratory tests, and treatment in children from three pediatric rheumatology centers in Poland.

## Materials and methods

Medical records of 27 patients with KD hospitalized at three pediatric rheumatology centers in Poland (Clinics of Pediatric Rheumatology in Wroclaw and Lublin Medical University, Department of Children Rheumatology in John Paul II Pediatric Center in Sosnowiec) between January 1, 2011, and June 31, 2012, were retrospectively reviewed. The study protocol was approved by the Ethics Committee of the Wroclaw Medical University.

Demographic features, diagnostic clinical features of KD, and additional clinical findings including arthritis and/or arthralgia, gastrointestinal symptoms, respiratory symptoms, and central nervous system symptoms were recorded for each patient. Moreover, all laboratory findings collected prior to IVIG administration were reviewed, including complete blood count (CBC) parameters, ESR, serum CRP, alanine transaminase (ALT), aspartate transaminase (AST), and albumin levels, urine analysis.

Electrocardiography (ECG), echocardiography, and cardiologist’s consultation of each patient were analyzed. Maximum coronary artery *z* scores were calculated for the right main coronary artery, the left main coronary artery, or the left anterior descending coronary artery. Patients were defined to have coronary artery abnormalities if their maximum coronary *z* score was >2.5 mm. They were further classified as small coronary artery aneurysms (CAA) if *z* score was between 2.5 and 5.0 mm, large if >5.0–10.0 mm, and giant if >10 mm.

The diagnosis of complete and incomplete KD was made using the AHA recommendations [7].

Each patient had a pediatrician’s and cardiologist’s appointment/assessment 6 months after he/she was discharged from the hospital.

### Statistical analysis

Data are expressed as median and range of continuous variables. Categorical data are expressed as absolute number and percentage. Comparison was made for all of the parameters between complete KD and incomplete KD as well as the whole group. The mean ranks between groups were compared using the Mann–Whitney *U* test. Categorical variables were compared using χ^2^ test with Yate’s correction or Fisher’s exact test for data continuity. To investigate the correlations between the variables, we used Pearson correlation. A *p* value <0.05 was considered to be statistically significant. A statistical analysis was performed using the EPI INFO Software version 3.5.2 (Centers for Disease Control and Prevention, Atlanta, USA, 2010).

## Results

Patients’ demographic, clinical, and laboratory features at the time of diagnosis are shown in Table 1. Among 27 Caucasian patients with KD (44 % males), 17 patients (63 %) had complete KD. The incidence of complete KD was higher in females (64.7 %) and incomplete KD in males (60 %), but these differences were not statistically significant.

**Table 1:** Demographic characteristic of the patients with Kawasaki disease

Parameter	Whole group *n* = 27 *n* (%)	Complete *n* = 17 *n* (%)	Incomplete *n* = 10 *n* (%)	*p*
Gender				
Male	12 (44.4)	6 (35.3)	6 (60.0)	NS
Female	15 (66.6)	11 (64.7)	4 (40.0)	
Age at diagnosis (months)				
<1	4 (14.8)	3 (17.6)	1 (10.0)	NS
1–9	21 (77.8)	12 (70.6)	9 (90.0)	
>9 years	2 (7.4)	2 (11.8)	–	
Weight, percentile				
<25	10 (37.0)	5 (29.4)	5 (50.0)	NS
25–75	9 (33.3)	6 (35.3)	3 (30.0)	
>75	6 (22.2)	6 (35.3)	–	
Seasonal onset				
Winter	5 (18.5)	3 (17.6)	2 (20.0)	NS
Springs	7 (25.9)	5 (29.4)	2 (20.0)	
Summer	8 (29.6)	4 (23.5)	4 (40.0)	
Autumn	4 (14.8)	2 (11.8)	2 (20.0)	

*NS* nonsignificant

The median age at disease onset was 3 years (range 4 months–12 years). Age did not differ significantly between patients with complete and incomplete KD.

The season of the onset of KD was winter in 18.5 %, spring in 25.9 %, summer in 29.6 %, and autumn in 14.8 % cases of the patients.

Clinical signs of patients with KD, complete and incomplete, at the time of diagnosis are presented in Table 2. All of the patients with complete KD presented polymorphous skin rash, oral mucosal changes, and bilateral nonpurulent conjunctivitis. Patients with incomplete KD compared with patients with complete KD significantly less presented cervical lymphadenopathy (20 vs. 88.2 %; *p* = 0.00075), changes in extremities (30 vs. 76.5 %; *p* = 0.04), and bilateral nonpurulent conjunctivitis (60 vs. 100 %; *p* = 0.01). Two (20 %) patients with incomplete KD presented two classical signs, and eight patients (80 %) presented three classical KD signs. There were also other symptoms presented by patients with KD (in the whole group): gastrointestinal symptoms (66.7 %), hepatic dysfunction (55.6 %), arthralgia and/or arthritis (44.4 %), and sterile pyuria (29.6 %). The differences between incomplete and complete KD were not significant.

**Table 2:** Classical and “nonclassical” symptoms in complete and incomplete KD

	Whole group *n* = 27 *n* (%)	Complete *n* = 17 *n* (%)	Incomplete *n* = 10 *n* (%)	*p*
Classic KD clinical symptoms				
Fever persisting at least 5 days	25 (92.6)	15 (88.2)	10 (100)	–
Bilateral conjunctival injection without exudates	23 (85.2)	17 (100)	6 (60.0)	0.012
Cervical lymphadenopathy (>1.5 cm diameter)	17 (62.9)	15 (88.2)	2 (20.0)	0.00075
Changes in lips and oral cavity	26 (96.3)	17 (100)	9 (90.0)	NS
Polymorphous exanthema	26 (96.3)	17 (100)	9 (90.0)	NS
Changes in extremities	16 (59.3)	13 (76.5)	3 (30.0)	0.040
2/3 classic KD clinical signs	–	–	2/8	
“Nonclassical” clinical symptoms				
Arthralgia, arthritis	12 (44.4)	8 (47.1)	4 (40.0)	NS
Gastrointestinal symptoms (diarrhea, vomiting, abdominal pain)	18 (66.7)	10 (58.8)	8 (80.0)	NS
Hepatic dysfunction	15 (55.6)	11 (64.7)	4 (40.0)	NS
Respiratory symptoms (cough)	4 (14.8)	4 (23.5)	–	NS
Sterile pyuria	8 (29.6)	6 (35.3)	2 (20.0)	NS

*KD* Kawasaki disease, *NS* nonsignificant

Analysis of laboratory investigations is presented in Table 3. We found no statistical differences in laboratory investigations between complete and incomplete KD.

**Table 3:** Laboratory investigations and cardiovascular findings at diagnosis in complete and incomplete KD

	Whole group *n* = 27	Complete *n* = 17	Incomplete *n* = 10	*p*
	Median (range)	Median (range)	Median (range)	
Laboratory investigations				
CRP (mg/l)	22.4 (3.7–225.6)	17.8 (3.7–179.9)	31.0 (5.8–225.6)	NS
CRP ≥ 3 + (*n*, %)	27 (100)	17 (100)	10 (100)	–
ESR (mm/h)	81.0 (15.0–130.0)	80.0 (28.0–112.0)	90.0 (15.0–.0)	NS
ESR ≥40 mm/h	23 (85.2 %)	15 (88.2 %)	8 (80.0 %)	NS
Red blood cells (×10^12^/l)	4.20 (3.05–4.5)	4.06 (3.05–4.54)	4.29 (3.17–4.75)	NS
Hematocrit (%)	32.2 (25.9–38.7)	31.2 (25.9–38.7)	35.7 (27.9–37.5)	NS
Hemoglobin (g/l)	11.2 (8.6–12.9)	10.9 (8.6–12.4)	11.9 (9.1–12.9)	NS
Anemia for age (*n*, %)	11 (40.7 %)	7 (41.2 %)	4 (40.0 %)	NS
White blood cells (× 10^9^/l)	14.6 (6.1–30.4)	14.6 (6.1–30.4)	14.9 (10.7–23.8)	NS
WBC count ≥15 × 10^9^/l (*n*, %)	13 (48.1	8 (47.1 %)	5 (50.0 %)	NS
Neutrophils (×10^9^/l)	12.0 (0.3–26.7)	12.4 (0.3–26.7)	8.6 (4.9–14.9)	NS
Platelets count (×10^9^/l)	643.0 (229.0–1121.0)	630.0 (229.0–1121.0)	689.5 (411.0–974.0)	NS
Platelets after 7 days ≥450 × 10^9^/l (*n*, %)	24 (88.8 %)	15 (88.2 %)	9 (90.0 %)	NS
Albumin (g/l)	3.27 (2.20–39.0)	3.27 (3.0–3.7)	3.41 (2.2–39.0)	NS
Albumin ≤3.0 g/dl (*n*, %)	22 (81.5 %)	17 (100 %)	5 (50.0 %)	NS
ALT (U/l)	30.0 (10.0–146.0)	28.5 (16.0–146.0)	45.0 (10.0–.0)	NS
Elevated ALT (*n*, %)	12 (44.45)	7 (43.8 %)	5 (50.0 %)	NS
AST (U/l)	44.0 (17.0–117.0)	42.0 (17.0–111.0)	49.0 (17.0–117.0)	NS
Elevated AST (*n*, %)	13 (48.1 %)	8 (47.1 %)	5 (50.0 %)	NS
Cardiovascular findings				
Coronary artery abnormalities				
*z* ≤ 2.5	15 (55.6 %)	12 (70.6 %)	6 (60.0 %)	NS
*z* 2.5–5.0	7 (25.9 %)	4 (23.5 %)	3 (30.0 %)	
*z* > 5.0–10.0	2 (7.4 %)	1 (5.9 %)	1 (10.0 %)	
Repolarization disorders	11 (64.7 %)	5 (29.4 %)	6 (60.0 %)	NS
Congestive heart failure, myocarditis, pericarditis, valvular regurgitation	4 (14.8 %)	4 (23.5 %)	–	NS

*KD* Kawasaki disease, *NS* nonsignificant

Cardiac assessments showed that the majority of patients with KD (whole group 55.6 %; complete KD 70.6 %, incomplete KD 60 %) had not developed coronary artery abnormalities (Table 3). The small CAA were noted in seven (25.9 %) patients, four (23.5 %) cases with complete KD and three (30 %) with incomplete KD. The large CAA were found in two patients, one (10 %) with complete KD and one (7.4 %) with incomplete KD. On ECGs the repolarization abnormalities were more often detected in patients with incomplete KD (60 %) compared to patients with complete KD (29.4 %); this difference was not statistically significant. Moreover, repolarization abnormalities were found in all cases with complete KD but without coronary artery abnormalities. In complete KD, congestive heart failure and/or myocarditis was presented in four (23.5 %) patients.

Analysis of patients’ medical records revealed that median time from first symptoms to KD diagnosis was 8.5 days (range 5–14 days) (Table 4). Despite the fact that the children with incomplete KD were diagnosed later (median time 11 days, range 5–14 days) than the children with complete KD (median time 7.50 days, range 5–13 days), this difference was not significant. The median time of hospitalization was 16.5 days (range 7–45 days) and did not significantly differ between patients with complete and incomplete KD.

**Table 4:** Clinical history in complete and incomplete KD

	Whole group, *n* = 27	Complete, *n* = 17	Incomplete, *n* = 10	*p*
	Median (range)	Median (range)	Median (range)	
Interval symptoms onset diagnosis, days	8.5 (5–14)	7.5 (5–13)	11 (5–14)	NS
Length of hospitalization, days	16.5 (7–45)	17 (7–45)	14 (9–24)	NS
Treated <10 days after symptoms onset, *n* (%)	16 (59.2 %)	13 (76.5 %)	3 (30 %)	0.026^a^

*KD* Kawasaki disease, *NS* nonsignificant

^a^Fisher’s exact test

We investigated the clinical symptoms and laboratory results to find factors associated with the duration of fever and hospitalization. We found significant negative correlation between RBC count (*r* = −0.55, *p* = 0.017), Ht (*r* = −0.56, *p* = 0.016), Hb (*r* = −0.58, *p* = 0.011), and platelet count (*r* = −0.60, *p* = 0.009) and positive correlation between WBC count (*r* = 0.72, *p* = 0.001), neutrophil count (*r* = 0.73, *p* = 0.001), and the length of hospitalization (data not shown). Other parameters correlated neither with the duration of hospitalization nor with fever.

We found no statistical significant correlations between the presence of coronary artery abnormalities and clinical symptoms and results of laboratory tests (data not shown).

All patients were treated with IVIG at 2 g/kg together with aspirin at the time of established diagnosis. Two (7 %) patients required retreatment with IVIG. Intravenous methylprednisolone was given in one case. In our study group, IVIG was administrated significantly less before the 10th day of disease in patients with incomplete KD, compared to patients with complete KD (*p* = 0.026) (Table 4).

Six months after discharge, an examination revealed that despite early treatment and three times IVIG retreatment, and three pulses of steroids, CAA had developed in one patient with complete KD and in one patient with incomplete KD (7 % of the whole group).

No mortality and recurrence were seen in the study group.

Moreover, during the 6 months, five (18.5 %) patients with KD had recurrent upper respiratory tract infections, one (3.7 %) patient had been diagnosed with transient hypoimmunoglobulinemia G and M of infants, and one patient (3.7 %) had been diagnosed with idiopathic juvenile arthritis.

## Discussion

The incidence of KD is increasing worldwide [5]. No study reporting incidence of KD has been published from Poland. In our study, 27 Caucasian children with KD were hospitalized in three pediatric rheumatology centers in Poland from January 2011 to June 2012. Incomplete KD was diagnosed in 37 % of children, which is consistent with other studies [10–12]. This proportion is higher than the 20 % reported in the previous studies [13, 14]. Published AHA guidelines, including supplementary laboratory criteria as well as the use of echocardiography, result in better recognition of KD [15].

Our study showed no statistically significant differences in the patients’ age between the complete and incomplete KD groups. This finding is not consistent with earlier studies. Other data suggest that incomplete KD is more common in children younger than 1 year of age and over eight [16, 17].

In our study, we analyzed the frequency of clinical signs. Of the five cardinal symptoms, conjunctival injection, mucosal changes, and polymorphous rash occurred most frequently. This is consistent with the results reported by other authors [18]. Patients with incomplete KD presented conjunctival injection, changes in extremities, and cervical lymphadenopathy significantly less. This distribution of clinical signs was reported in the previous studies [10, 14].

Except for classical clinical signs, other clinical symptoms of KD have been reported previously [19]. In our study, we found the following “nonclassical” symptoms of KD: cough, abdominal pain, vomiting, diarrhea, and arthralgia, which is similar to what was reported in the previous studies [14, 19].

Published studies have shown that incomplete KD is associated with higher risk of developing coronary abnormalities [13, 20]. We did not find any differences in CAA incidence between complete KD and incomplete KD either at the time of diagnosis or after 6 months of follow-up. Interestingly, although we did not find CAA in incomplete KD children on echocardiography, in most cases ECG revealed depolarization disturbances. This finding may suggest ECG examination as a more sensitive test to recognize cardiac involvement in the acute phase of KD, which was shown in the previous reports [21, 22]. Further studies are needed to confirm this.

We also observed correlation between low RBC, Hb concentration, Ht values, platelet count, elevated WBC, neutrophil count, and duration of hospitalization. This may reflect severity of the disease. Printz et al. [23] reported the relationship between elevated WBC count and coronary cardiac abnormalities. Furthermore, Falcini et al. [18] suggested that unexplained febrile child with elevated acute-phase reactants and platelet count requires echocardiography assessment.

Optimal time for the IVIG therapy is within 7–10 days of illness [7]. The data have shown that delayed IVIG administration due to delayed incomplete KD diagnosis increases the risk of developing CAA [24]. Although our results showed that patients with incomplete KD were treated with IVIG later than within the first 10 days of disease, there were no differences in the prevalence of CAA between the two groups.

In our study, its retrospective nature and the small size of the sample are the main limitations. Because of this, our findings are rather suggestive and not definitive.

In conclusion, we found no differences in demographic features, age of onset, and laboratory tests of patients with complete KD and incomplete KD. Incomplete KD may therefore be characterized by less frequent of cervical lymphadenopathy, changes in extremities, and bilateral nonpurulent conjunctivitis. High WBC and anemia may suggest a more severe course of disease. The ventricular repolarization disturbances in ECG of children with incomplete KD without coronary abnormalities may be the only manifestation of myocardial involvement. Despite the fact that incomplete forms of KD presentation often delay diagnosis, in most patients treatment with IVIG can help avoid complication of CAA.

## References

[1] Nakamura Y, Yashiro M, Uehara R, Sadakane A, Tsuboi S, Aoyama Y, Kotani K, Tsogzolbaatar EO, Yanagawa H (2012). Epidemiologic features of Kawasaki disease in Japan: results of the 2009–2010 Nationwide Survey. J Epidemiol.

[2] Holman RC, Belay ED, Christensen KY, Folkema AM, Steiner CA, Schonberger LB (2010). Hospitalizations for Kawasaki syndrome among children in the United States, 1997–2007. Pediatr Infect Dis J.

[3] Heuclin T, Dubos F, Hue V, Godart F, Francart C, Vincent P (2009). Hospital network for evaluating the management of common childhood diseases, Martinot A. increased detection rate of Kawasaki disease using new diagnostic algorithm, including early use of echocardiography. J Pediatr.

[4] Salo E, Griffiths EP, Farstad T, Schiller B, Nakamura Y, Yashiro M, Uehara R, Best BM, Burns JC (2012). Incidence of Kawasaki disease in northern European countries. Pediatr Int.

[5] Fischer TK, Holman RC, Yorita KL, Belay ED, Melbye M, Koch A (2007). Kawasaki syndrome in Denmark. Pediatr Infect Dis J.

[6] Schiller B, Fasth A, Bjorkhem G, Elinder G (1995). Kawasaki disease in Sweden: incidence and clinical features. Acta Paediatr.

[7] Newburger JW, Takahashi M, Gerber MA, Gewitz MH, Tani LY, Burns JC, Shulman ST, Bolger AF, Ferrieri P, Baltimore RS, Wilson WR, Baddour LM, Levison ME, Pallasch TJ, Falace DA, Taubert KA, Committee on Rheumatic Fever, Endocarditis and Kawasaki Disease, Council on Cardiovascular Disease in the Young; American Heart Association, American Academy of Pediatrics (2004). Diagnosis, treatment, and long-term management of Kawasaki disease: a statement for health professionals from the committee on rheumatic fever, Endocarditis and Kawasaki disease, council on cardiovascular disease in the young, American heart association. Circulation.

[8] Tremoulet AH, Jain S, Chandrasekar D, Sun X, Sato Y, Burns JC (2011). Evolution of laboratory values in patients with Kawasaki disease. Pediatr Infect Dis J.

[9] Uehara R, Yashiro M, Oki I, Nakamura Y, Yanagawa H (2007). Re-treatment regimens for acute stage of Kawasaki disease patients who failed to respond to initial intravenous immunoglobulin therapy: analysis from the 17th nationwide survey. Pediatr Int.

[10] Lin YT, Manlhiot C, Ching JC, Han RK, Nield LE, Dillenburg R, Pepelassis D, Lai LS, Smythe JF, Chahal N, Yeung RS, McCrindle BW (2010). Repeated systematic surveillance of Kawasaki disease in Ontario from 1995 to 2006. Pediatr Int.

[11] Corinaldesi E, Fabi M, Capra L, Landini Ch, Marino F (2012). A retrospective study of Kawasaki disease in a cohort of patients from Emilia Romagna, Italy, 2000–2010. Pediatr Int.

[12] Al-Ammouri I, Al-Wahsh S, Khuri-Bulos N (2012). Kawasaki disease in Jordan: demographics, presentation, and outcome. Cardiol Young.

[13] Burns JC, Wiggins JW, Toews WH, Newburger JW, Leung DY, Wilson H, Glodé MP (1986). Clinical spectrum of Kawasaki disease in infants younger than 6 months of age. J Pediatr.

[14] Manlhiot C, Christie E, McCrindle BW, Rosenberg H, Chahal N, Yeung RS (2012). Complete and incomplete Kawasaki disease: two sides of the same coin. Eur J Pediatr.

[15] Ghelani SJ, Sable C, Wiedermann BL, Spurney CF (2012). Increased incidence of incomplete Kawasaki disease at a pediatric hospital after publication of the 2004 American heart association guidelines. Pediatr Cardiol.

[16] Song D, Yeo Y, Ha K, Jang G, Lee J, Lee K, Son C, Lee J (2009). Risk factors for Kawasaki disease-associated coronary abnormalities differ depending on age. Eur J Pediatr.

[17] Yeo Y, Kim T, Ha K, Jang G, Lee J, Lee K, Son C, Lee J (2009). Incomplete Kawasaki disease in patients younger than 1 year of age: a possible inherent risk factor. Eur J Pediatr.

[18] Falcini F, Ozen S, Magni-Manzoni S, Candelli M, Ricci L, Martini G, Cuttica RJ, Oliveira S, Calabri GB, Zulian F, Pistorio A, La Torre F, Rigante D (2012). Discrimination between incomplete and atypical Kawasaki syndrome versus other febrile diseases in childhood: results from an international registry-based study. Clin Exp Rheumatol.

[19] Yun SH, Yang NR, Park SA (2011). Associated symptoms of Kawasaki disease. Korean Circ J.

[20] Ha KS, Jang G, Lee J, Lee K, Hong Y, Son C, Lee J (2012). Incomplete clinical manifestation as a risk factor for coronary artery abnormalities in Kawasaki disease: a meta-analysis. Eur J Pediatr.

[21] Crystal MA, Syan SK, Yeung RSM, Dipchand AI, McCrindle BW (2008). Echocardiographic and electrocardiographic trends in children with acute Kawasaki disease. Can J Cardiol.

[22] Kuriki M, Fujino M, Tanaka K, Horio K, Kusuki H, Hosoi M, Eryu Y, Kato T, Yamazaki T, Hata T (2011). Ventricular repolarization lability in children with Kawasaki disease. Pediatr Cardiol.

[23] Printz BF, Sleeper LA, Newburger JW, Minich LL, Bradley T, Cohen MS, Frank D, Li JS, Margossian R, Shirali G, Takahashi M, Colan SD (2011). Pediatric heart network investigators. Noncoronary cardiac abnormalities are associated with coronary artery dilation and with laboratory inflammatory markers in acute Kawasaki disease. J Am Coll Cardiol.

[24] Sudo D, Monobe Y, Yashiro M, Mieno MN, Uehara R, Tsuchiya K, Sonobe T, Nakamura Y (2012). Coronary artery lesions of incomplete Kawasaki disease: a nationwide survey in Japan. Eur J Pediatr.

